# Successful Surgical Therapy of a Double Aortic Arch in a 10-Month-Old Mixed Breed Dog

**DOI:** 10.1155/2019/6519041

**Published:** 2019-02-13

**Authors:** Christelle M. Follette, Alex Terreros, Sheldon L. Padgett

**Affiliations:** Department of Surgery and Neurology, Metropolitan Veterinary Hospital, 1053 S. Cleveland-Massillon Road, Akron, OH, 44321, USA

## Abstract

A 10-month-old female spayed mixed breed dog with a suspected vascular ring anomaly was presented for exercise intolerance and wheezing. Computed tomography (CT) revealed a double aortic arch. The smaller right aortic arch was successfully ligated via right 4th intercostal thoracotomy. The patient was discharged one day postoperatively and continued to have good outcome at recheck 3.5 weeks after surgery. This is the 4th documented case of double aortic arch with a successful outcome. Preoperative CT scan was vital in preoperative planning and should be strongly recommended in all cases of suspected vascular ring anomalies with atypical presentation.

## 1. Introduction

Vascular ring anomalies are the result of abnormal development of aortic arches leading to entrapment of the esophagus and trachea by a complete or incomplete ring of vessels. During normal embryonic development, 6 pairs of aortic arches undergo selective involution and reconnection to form the great vessels, with the left 4th aortic arch eventually becoming the adult aortic arch and the right 4th aortic arch partially regressing to become the right subclavian artery. There are several varieties of vascular ring anomalies; however, 95% percent of dogs with clinical signs have a persistent right aortic arch (PRAA) with a left ligamentum arteriosum [1]. Clinical signs often involve regurgitation after eating solid foods, which is frequently observed in young animals at weaning [2, 3]. Double aortic arch results from persistence of both left and right fourth aortic arches and can cause compression of both the esophagus and trachea [4–6]. In humans, though vascular rings consist of less than 1% of cardiovascular malformations [7], double aortic arch is one of the most common, representing over 50% of complete vascular rings that undergo surgical correction [8, 9]. CT or MRI is currently the preferred imaging modalities for preoperative planning [10–13]. Double aortic arch in dogs is considered rare with very few reports published in the literature [4, 6, 14–22]. It has also been reported in a Siamese cat [23], a talapoin monkey [24], and a white lion [25].

## 2. Case Description

**Figure 1:**
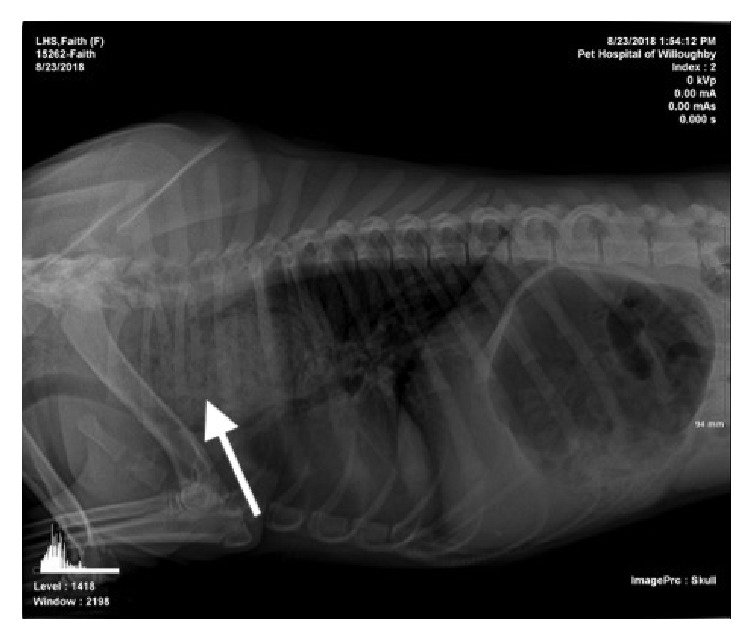
Referral right lateral thoracic radiograph showing cranial esophageal dilation with food (white arrow).

A 10-month-old, female spayed Labrador retriever mix was referred to Metropolitan Veterinary Hospital Surgery Service due to a history of exercise intolerance and wheezing that was first recorded after being taken in by a local humane society. Radiographs from the referring veterinarian showed significant dilation of the cranial esophagus containing what appeared to be food as well as evidence of aspiration pneumonia (Figure 1). A contrast esophagram was performed by the same veterinarian subsequently and this examination was suggestive of a vascular ring anomaly. Despite these findings, the dog did not have a history of any episodes of vomiting or regurgitation during the 4 weeks of observation at the humane society. The dog was receiving amoxicillin-clavulanic acid 250 mg PO q12h (Clavamox; Zoetis, Kalamazoo, MI, USA) and ciprofloxacin 125 mg PO q12h (Cipro; Bayer Inc., Mississauga, Ontario, Canada) when initially evaluated at Metropolitan Veterinary Hospital.

Initial physical examination revealed no signs of dyspnea; however lower respiratory stridor, suggestive of stricture, was auscultated during panting. The dog was bright and alert, normothermic, and had no evidence of other congenital anomalies. The remainder of the physical examination was unremarkable. Likely differential diagnoses for the cranial esophageal dilation included vascular ring anomaly, or less likely esophageal stricture, foreign body, or neoplasia at the level of the heart base.

**Figure 2:**
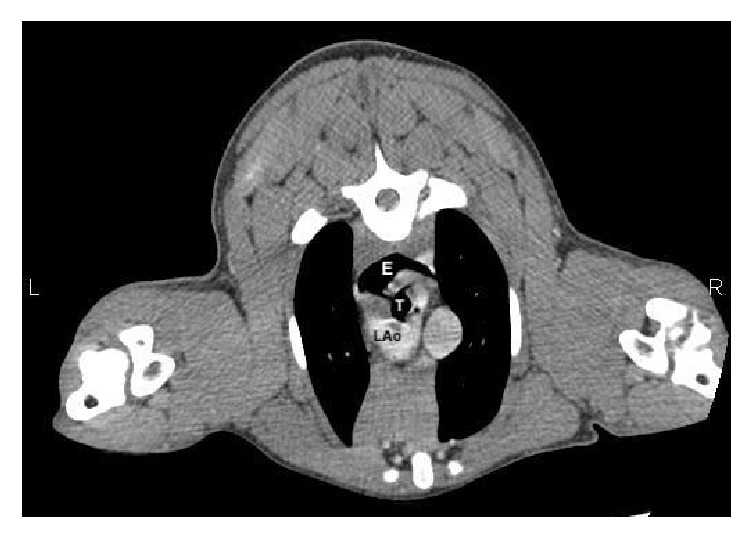
Post-contrast computed tomography (CT) angiogram with contrast showing compression of the trachea (T) by the smaller aberrant right aortic arch (black asterisk) and esophageal dilation (E). Note also the origin of the left aortic arch in the normal position (LAo).

Because of the atypical history mostly related to respiratory signs and the older age of the patient, advanced imaging was recommended for surgical planning prior to the procedure. Computed tomography (CT) evaluation (Picker PQS Third Generation CT Scanner; Coral Springs, FL, USA) of the thorax identified a segmental megaesophagus extending throughout the cervical and cranial thoracic region with termination of dilation at the level of the heart base (Figure 2). At this level, the aortic arch had a bifid course with a large left-sided normal branch and a smaller right-sided segment (approximately one half the size as the left arch) coursing from the level of the cranial aspect of the aortic arch and anastomosing with the proximal aspect of the descending aorta. The smaller right-sided branch encircled the trachea causing severe mediolateral luminal narrowing of the trachea at the level of the cranial most aspect of the aortic arch and was also encircling the esophagus at the level of the heart base. The esophagus was also compressed and bounded dorsolaterally on the left by the normal left-sided aortic arch, laterally on the right by the trachea and ventrally by the main pulmonary artery. The esophagus caudal to the vascular anomaly was minimally distended. The left subclavian artery arose from the normal left aortic arch and there was a lack of a normal brachiocephalic trunk. The right subclavian artery arose from the aberrant right aortic arch and there was a very short bi-carotid trunk that arose from the confluence of the aortic arch cranially. The pulmonary parenchyma was normal with no evidence of aspiration pneumonia or increased attenuation to indicate fibrosis from a previous aspiration. The remaining intrathoracic structures and the captured intra-abdominal structures were normal.

Diagnosis consisted of a double aortic arch (smaller right-sided aberrant branch and normal left-sided aortic arch) encircling both the esophagus and trachea resulting in compression of both of these structures at the level of the heart base with subsequent development of a segmental megaesophagus. Concurrent vascular anomalies resulting in atypical origins of the right subclavian and carotid arteries were also present.

Four weeks after performing the CT scan, the patient was admitted to the hospital for surgical correction of her double aortic arch. She was placed under general anesthesia for surgery with a standard protocol. She was premedicated with hydromorphone (Akorn, Inc., Lake Forest, IL, USA) 0.1 mg/kg body weight (BW), IM, and induced with diazepam (Hospira, Inc., Lake Forest, IL, USA) 0.25 mg/kg body weight (BW), IV, and propofol (PropoFlo28; Zoetis, Kalamazoo, MI, USA) 4 mg/kg body weight (BW), IV. General anesthesia was maintained with isoflurane (IsoSol; VEDCO, Inc., St. Joseph, MO, USA) and mechanical ventilation (Hallowell EMC Veterinary Anesthesia Ventilator Model 2002IEPro; Pittsfield, MA, USA) was provided throughout the procedure. Antibiotic therapy (Cefazolin; West-Ward Pharmaceutical Corp, Eatontown, NJ, USA), 22 mg/kg body weight (BW), IV, was administered every 90 min.

**Figure 3:**
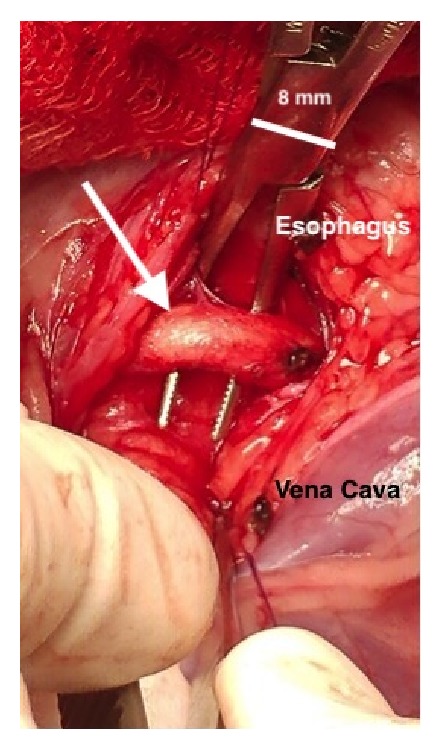
Aberrant right aortic arch seen via right 4th lateral thoracotomy (white arrow). The dilated esophagus can be seen at the top of the figure behind the right-angle forceps with the cranial vena cava at the bottom right.

**Figure 4:**
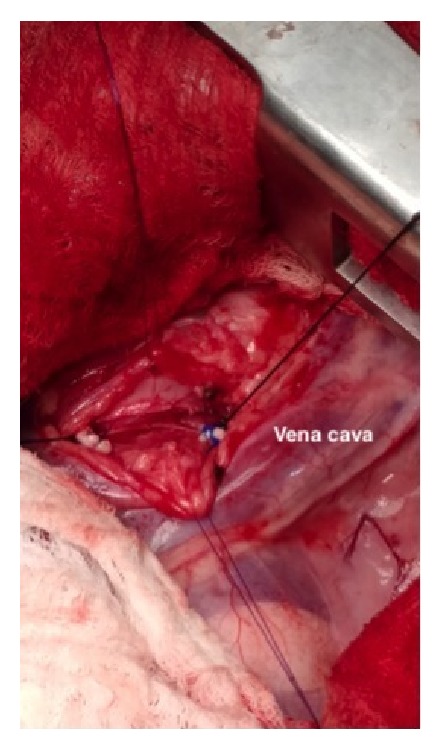
Posttransection of the right aortic arch with stay sutures in place. Note the vessel ends sutured with Prolene. The dog's head is to the right.

A routine right 4th intercostal lateral thoracotomy was performed. Upon completion of thoracic exploration, blunt dissection into the mediastinum was performed. Two nylon stay sutures (Ethilon; Ethicon, Inc., Somerville, NJ, USA) were placed for retraction of the mediastinum. No gross abnormalities were identified in the mediastinum other than the cranial megaesophagus and a 6 mm right aortic arch (Figure 3). Blunt dissection to isolate the aberrant vessel was performed. Temporary occlusion via vascular clamps for 10 minutes showed no change in heart rate or blood pressure. Ligation of the aortic arch was initially performed with 2-0 silk (Sofsilk; Covidien LLC., Mansfield, MA, USA) ligatures cranially and caudally. The vessel was then transected and each end was oversewn with 3-0 Prolene (Ethicon, Inc., Somerville, NJ, USA). Further dissection deep to the arch to the level of the esophagus was performed to ensure no obvious fibrous bands were causing further constriction (Figure 4). Orogastric intubation was performed and confirmed an appropriate diameter of the esophagus. Digital palpation of the trachea showed that constriction was significantly relieved. Routine thoracostomy tube placement was performed (Mila International, Inc., Florence, KY, USA). Routine thoracotomy closure and thoracostomy tube fixation was performed. Air was evacuated from the pleural cavity via the thoracostomy tube, restoring subatmospheric pressure.

The patient received an intercostal nerve block using bupivacaine liposome injectable solution (Nocita; Aratana Therapeutics, Inc., Leawood, KS, USA) intraoperatively and was maintained on continuous rate infusion (CRI) of fentanyl (Hospira, Inc., Lake Forest, IL, USA), initially. Upon transfer to the surgical theater, a technical error led to the patient receiving a substantially higher dose of fentanyl (64 mcg/kg bolus over 15 minutes) than planned (4 mcg/kg/hour). The infusion was discontinued for the remainder of the anesthesia. The patient developed sinus bradycardia following the administration of fentanyl and was treated with Atropine 0.02 mg/kg, IV (Med-Pharmex, Inc., Pomona, CA, USA) to which she responded adequately. Recovery from anesthesia was uneventful. Isoflurane was discontinued and the patient was extubated 10 minutes later. The patient was maintained on the same fluid supplementations until being transferred to the intensive care unit (ICU). The postoperative analgesic protocol also included a postoperative carprofen injection 4.4 mg/kg subcutaneously (Rimadyl; Zoetis, Kalamazoo, MI, USA) and fentanyl transdermal patch (Alvogen, Inc., Pine Brook, NJ, USA) 50 mcg/hour was placed on the dorsal cervical area.

On admission to the ICU, the patient was calm and comfortable and vitals were within normal range. Fentanyl administration at a dose of 4 mcg/kg/hour was reinstated 3 hours following extubation due to observed signs of discomfort on incisional palpation. From anesthetic recovery to discharge, the patient's clinical condition progressively improved. Parenteral medications were transitioned from CRI (Fentanyl 4 *μ*g/kg BW per hour CRI) to transdermal (Fentanyl patch) and oral nonsteroidal anti-inflammatory medication (Rimadyl; Zoetis, Kalamazoo, MI, USA), 75 mg PO q12h. The patient's thoracostomy tube air and fluid production decreased progressively during hospitalization and the tube was removed 12 hours postoperatively. The dog's first meal was offered 16 hours postoperatively as meatballs of CM (Hill's Pet Nutrition, Inc., Topeka, KS, USA) fed with the patient's head raised. No complications occurred during each subsequent feeding until discharge.

The dog was discharged one day postoperatively with instruction for strict exercise restriction for 3 weeks, oral Carprofen (Rimadyl), and transdermal fentanyl patch. Her caregivers at Lake Humane Society were instructed to feed small frequent meals until reevaluation.

A recheck was performed at Metropolitan Veterinary Hospital 3.5 weeks postoperatively. Her foster reported that she had resolution of her clinical signs starting one day after surgery. She was acting like a normal puppy, with great energy with no evidence of wheezing. After discharge postoperatively she was being fed one cup of food 3 times daily for 2 weeks. She was reported to have 2 episodes of regurgitation while on this feeding regimen. This resolved after switching her to 1.5 cups of solid dry dog food twice daily. At the time of recheck, on physical exam she was eupneic and no wheezing was auscultated. Three-view thoracic radiographs were performed which showed widening of the trachea cranial to the heart base, resolution of previous ventral tracheal deviation, and static cranial esophageal dilation. She had continued to do well with twice daily feeding from a bowl on the floor. No elevated feeding had been required.

## 3. Discussion

The typical presentation of canine patients with vascular ring anomalies involves a young patient with regurgitation immediately postweaning [2, 3]. This is the result of a complete vascular ring encircling the esophagus leading to cranial esophageal dilation [6]. With double aortic arch, dyspnea, wheezing, and other respiratory signs may also be seen [4, 20–22, 26]. Indeed, human patients with vascular ring anomalies have a higher incidence of respiratory signs (70-95%) than gastrointestinal signs (5-50%) [27, 28]. In this patient, wheezing and exercise intolerance were the only clinical signs observed despite the significant cranial esophageal dilation visible on thoracic radiographs.

In humans, vascular rings are classified by the International Congenital Heart Surgery Nomenclature and Database committee [29]. Double aortic arches are categorized into 3 subcategories: right arch dominant, left arch dominant, and balanced arches. The right aortic arch is most commonly the dominant arch, followed by left dominant, and symmetrical aortic arches are the least common [8]. All 3 categories have been reported in the veterinary literature [4, 6, 19–22]. In this case, unlike the majority of humans, the dominant aorta was on the left, making the right aortic arch the better candidate for ligation. Knowing which arch is dominant is vital for appropriate surgical planning and treatment of double aortic arch.

While thoracic radiographs and esophogram are often sufficient to diagnose a suspected vascular ring anomaly, they do not provide specific information as to the configuration of the vascular ring. Advanced imaging modalities that can aid in surgical planning include contrast angiography, echocardiography, magnetic resonance imaging (MRI), and computed tomography (CT). While contrast angiography can give an accurate visualization of the aortic arches and even identify the dominant arch in cases of double aortic arch [30], it has the disadvantage of being two-dimensional as well as invasive and technically challenging. Echocardiography is used in humans with vascular ring anomalies, especially neonates [31], but has a limited use in the detection of extracardiac structures and a small acoustic window. For these reasons, it is not frequently used in veterinary patients [32]. Both MRI and CT have the advantage of being able to provide a three-dimensional view of the great vessels and are the imaging modalities of choice in human patients with suspected vascular rings [33]. In humans, MRI has been advocated as most appropriate first-line imaging for patients suspected to have vascular rings [34, 35]. Disadvantages of MRI include availability, cost, and the need for multiple sequences which require sedation (in humans) or general anesthesia (in veterinary medicine). Any or all of these factors prohibit the frequent use of MRI for diagnosis of vascular ring anomalies in veterinary medicine. CT is commonly used in human medicine and the preferred imaging modality in many institutions due to the detailed depiction of the great vessel anatomy and shorter scanning time that does not require sedation [8, 36]. While in veterinary medicine general anesthesia is still often required for precise imaging, CT is overall more readily available than MRI and offers a 3D view of the vascular ring to allow for accurate surgical planning.

PRAA with a left ligamentum arteriosum is present in 95% of canine patients with vascular ring anomalies [1]. Therefore, in a patient with typical presentation, it is not unreasonable to proceed with a thoracic exploration via left fourth thoracotomy without an actual diagnosis using 3D imaging or angiography. This is often the case in veterinary medicine, as in a recent study of PRAA only 2/30 (7%) of cases had a CT with triple-phase angiography performed for preoperative planning [37]. However, this puts the surgeon at a disadvantage in the 5% of the time that an uncommon vascular ring anomaly is present. Indeed, in the previously reported literature [4, 6, 14–22], the diagnosis of double aortic arch was only made intraoperatively or at necropsy. The atypical presentation of an older patient with respiratory signs led the authors to more strongly recommend CT prior to exploratory thoracotomy. In this particular case, the CT scan was able to allow direct visualization of the double aortic arch and planning of the surgical approach based on the nondominant right aortic arch. To our knowledge, this is the only case of double aortic arch in the literature that had an advanced imaging diagnosis prior to surgery. In previous cases of surgical correction of double aortic arch, a good outcome was achieved despite the lack of surgical planning [19, 21, 22]. However, the addition of CT imaging may have contributed to the excellent outcome in this case, as it allowed planning for a right-sided thoracotomy which led to less unnecessary dissection that may have been sustained if a standard left thoracotomy approach had been made.

Prognosis for dogs undergoing correction of vascular ring anomalies is variable. In some cases, relieving the constriction of the esophagus is curative and the cranial esophageal dilation regresses with time [2]. However, in other cases regurgitation can persist [2]. Prognosis for double aortic arch correction has historically been regarded as poor. No dogs had been reported to survive surgical correction of a double aortic arch until a 3-month-old mixed breed dog in 2004 [19]. Since then there have been two other reported cases of a 7-week-old German Shepherd [21] and a 10-week-old Czechoslovakian wolfdog [22] who survived to be discharged from the hospital. The patient presented here is the 4th patient reported in the literature to have a good outcome from surgery. Previous cases who have survived surgery have gone on to have a good long-term outcome and resolution of clinical signs, as was also true in the case presented here. Therefore, though there are risks associated with double aortic arch attenuation, a good clinical outcome is possible.

In conclusion, in suspected cases of vascular ring anomalies, atypical presentation or the presence of unusual clinical signs should prompt clinicians to more strongly recommend advanced imaging (such as CT angiogram) to aid in surgical planning. Good clinical outcome is possible after attenuation of double aortic arch if the patient survives the perioperative period.
